# REFINE (REduced Frequency ImmuNE checkpoint inhibition in cancers): A multi-arm phase II basket trial testing reduced intensity immunotherapy across different cancers

**DOI:** 10.1016/j.cct.2022.107030

**Published:** 2022-11-26

**Authors:** Sophie Merrick, Matthew Nankivell, Matteo Quartagno, Caroline S. Clarke, Nalinie Joharatnam-Hogan, Tom Waddell, Brent O'Carrigan, Michael Seckl, Ehsan Ghorani, Emma Banks, Kim Edmonds, George Bray, Rose Woodward, Rachel Bennett, Jonathan Badrock, Will Hudson, Ruth E. Langley, Naveen Vasudev, Lisa Pickering, Duncan C. Gilbert

**Affiliations:** a MRC Clinical Trials Unit at UCL, Institute of Clinical Trials & Methodology, 2nd Floor 90 High Holborn, London WC1V 6LJ, UK; b University College London (UCL) Research Department of Primary Care and Population Health, Upper 3rd Floor, UCL Medical School, Royal Free Campus, London NW3 2PF, UK; c The Christie NHS Foundation Trust, Wilmslow Road, Manchester M20 4BX, UK; d Department of Oncology, Cambridge University Hospitals NHS Foundation Trust, Addenbrooke's Hospital, Cambridge Biomedical Campus Hill's Road, Cambridge CB2 0QQ, UK; e Imperial College London, Division of Cancer, 2nd floor U201 Hammersmith Hospital Campus, London W12 0NN, UK; f Leeds Teaching Hospitals NHS Trust, Beckett Street, Leeds LS9 7TF, UK; g Royal Marsden Hospital, 203 Fulham Rd, Chelsea, London SW3 6JJ, UK; h Action Kidney Cancer, 11th Floor, 3 Piccadilly Place, Manchester M1 3BN, UK

**Keywords:** REFINE, Renal cancer, Immune checkpoint inhibitor, MAMS, Platform trial

## Abstract

**Background:**

Immune checkpoint inhibitors (ICI) have revolutionised treating advanced cancers. ICI are administered intravenously every 2–6 weeks for up to 2 years, until cancer progression/unacceptable toxicity. Physiological efficacy is observed at lower doses than those used as standard of care (SOC). Pharmacodynamic studies indicate sustained target occupancy, despite a pharmacological half-life of 2–3 weeks. Reducing frequency of administration may be possible without compromising outcomes. The REFINE trial aims to limit individual patient exposure to ICI whilst maintaining efficacy, with potential benefits in quality of life and reduced drug treatment/attendance costs.

**Methods/Design:**

REFINE is a randomised phase II, multi-arm, multi-stage (MAMS) adaptive basket trial investigating extended interval administration of ICIs. Eligible patients are those responding to conventionally dosed ICI at 12 weeks. In stage I, patients (n = 160 per tumour-specific cohort) will be randomly allocated (1:1) to receive maintenance ICI at SOC vs extended dose interval. REFINE is currently recruiting UK patients with locally advanced or metastatic renal cell carcinoma (RCC) who have tolerated and responded to initial nivolumab/ipilimumab, randomised to receive maintenance nivolumab SOC (480 mg 4 weekly) vs extended interval (480 mg 8 weekly). Additional tumour cohorts are planned. Subject to satisfactory outcomes (progression-free survival) stage II will investigate up to 5 different treatment intervals. Secondary outcome measures include overall survival, quality-of-life, treatment-related toxicity, mean incremental pathway costs and quality-adjusted life-years per patient. REFINE is funded by the Jon Moulton Charity Trust and Medical Research Council, sponsored by University College London (UCL), and coordinated by the MRC CTU at UCL.

Trial Registration

ISRCTN79455488.

NCT04913025

EUDRACT #: 2021-002060-47.

CTA 31330/0008/001-0001;

## 1 Introduction

Immune checkpoint inhibitors (ICI) have revolutionised cancer treatment over the last decade, resulting in durable responses and long-term survival in several cancer types [1–3]. ICI are monoclonal antibodies that target immune checkpoints including programmed cell death 1 receptor (PD-1) and its ligand 1 (PD-L1), which play a key role in maintaining immune homeostasis. Inhibition of these checkpoints restores the ability of the immune system to recognise and destroy cancer cells.

Despite their success, ICI impose a significant burden on patients and healthcare systems. ICI are routinely administered on a continuous schedule of frequent intravenous administrations (currently every 2–6 weeks) until cancer progression or the development of unacceptable toxicity. This approach requires a major commitment of resources for individual patients and health services alike. Disappointingly, due to the financial burden associated with treatment, ICI are not available globally to many who might benefit [4].

At present, there is limited understanding around predictive biomarkers for initial use or continued treatment with ICI, resulting in significant over treatment [5,6]. This increases treatment toxicity, negatively impacts quality of life (QOL) and has a significant and potentially avoidable financial impact on healthcare services.

Physiological efficacy of ICI (measured by PD-1 T cell receptor occupancy) is observed at much lower doses than those that have become standard of care (SOC) [7] and pharmacodynamic studies indicate sustained target occupancy on T cells of >70% for at least 2 months despite a pharmacological half-life of 2–3 weeks [8]. Furthermore, it has been observed that the rate of clearance of ICI decreases in patients responding to treatment as their disease status improves [9]. The potential prolonged physiological activity of these drugs means that as an alternative to early cessation, extending the interval of administration (i. e. reducing the frequency) should be possible, without compromising outcomes. This is supported by simulation analyses for pembrolizumab and nivolumab [10].

A number of clinical trials are currently testing early cessation in patients experiencing clinical response, either at a pre-determined timepoint such as 1 year (DANTE in melanoma or SAVE in non-small cell lung cancer [11,12]) or at maximal clinical response (SAFE-STOP and PET-STOP, in melanoma [13,14]).

REFINE (REduced Frequency ImmuNE checkpoint inhibition in cancers) is a clinical trial using a multistage approach to test whether it is possible to extend the interval between treatments in patients with metastatic cancer that are responding to ICI treatment without significantly losing efficacy [15]. There is a strong biologic rationale, coupled with potential major benefits including reduced treatment toxicity, improved QOL and reduced healthcare resource use and costs. REFINE includes a health economic component to evaluate the cost-effectiveness of extended interval treatment with ICI. Reducing costs and the burden associated with treatment may markedly improve access to ICI globally allowing populations to benefit from their transformative cancer outcomes.

## 2 Methods

### 2.1 Overview of design

**Figure 1:**
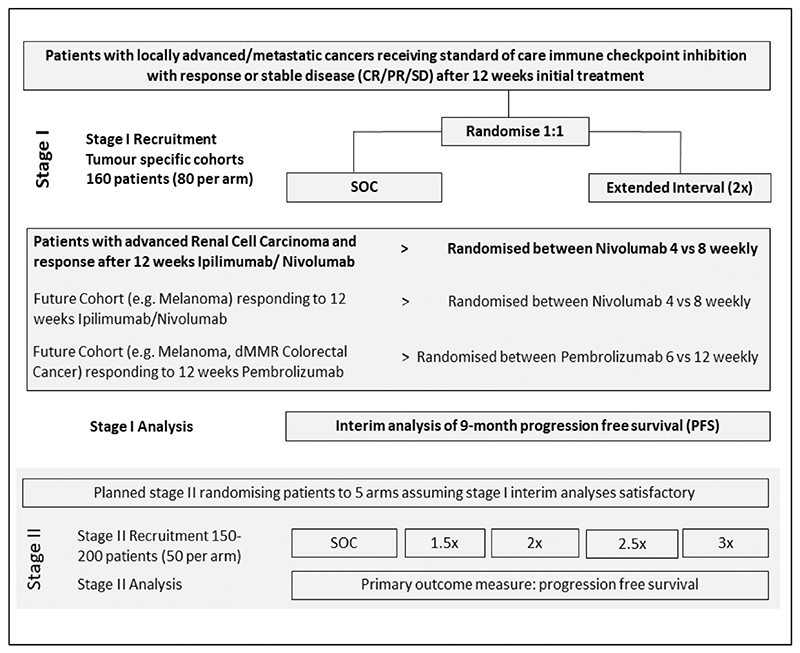
REFINE trial schema.

REFINE is a phase II, multi-arm multi-stage (MAMS) basket trial investigating extended interval administration of ICI across multiple cancer types. It is a preliminary step towards a DURATIONS trial [16,17] using a MAMS approach to ultimately define a dosing interval-response curve and identify the optimal treatment schedule for ICI in patients with advanced cancer. Eligible patients should have an initial (radiological) response to ICI at 12 weeks. In stage I, patients (*n* = 160 per tumour-specific cohort) will be randomly allocated (1:1) to receive ongoing ICI as per SOC versus at extended dosing interval. The initial cohort (currently recruiting) is patients with locally advanced (inoperable) or metastatic renal cell carcinoma (RCC) who have received SOC treatment with nivolumab and ipilimumab. Responding patients (complete or partial response or stable disease on their 12-week CT scan) are randomly allocated to receive maintenance nivolumab as SOC (480 mg 4 weekly) versus extended interval dosing (480 mg 8 weekly). The next cohort to open will be melanoma and it is intended that subsequent cohorts (such as MSI high colorectal cancer) will be added in the next 12 months (Fig. 1).

Interim results will be analysed approximately 6 months from completion of stage I recruitment when the majority of patients will have had a least 9 months on trial (i.e. 1 year after starting ICI). These results will inform stage II which will investigate up to 5 different treatment intervals in subsequently recruited patients. An amendment will be submitted to the MHRA for approval prior to the commencement of the second stage.

Completion of the first two stages of REFINE will demonstrate the feasibility of recruiting to a phase III trial testing extended dosing interval of administration of different ICI in responding patients across multiple indications.

### 2.2 Outcome measures

The primary outcome measure in REFINE is progression free survival (PFS). PFS is defined as the interval from randomisation to first evidence of local recurrence, new primary cancer, distant metastases (new or progression), or death from any cause, whichever occurs first. Analysis will be performed approximately 6 months after stage I recruitment has completed and will inform the design of the second stage.

The following secondary outcome measures will be analysed:

- Overall survival (OS)
- Quality of Life (QoL) (EORTC QLQ-C30 and EQ-5D-5L)
- Treatment-related toxicity
- Mean incremental cost per patient
- Mean incremental QALYs per patient (calculated from EQ-5D-5L)
- Cost-utility analysis assessing cost-effectiveness of reduced versus standard frequency administration
- Feasibility of recruitment to each cohort

### 2.3 Sample size

REFINE will enrol 160 patients into stage I for each cohort (80 patients randomised to each arm). This will provide 80% power at a 5% one-sided significance level to detect an absolute reduction of 20 percentage points in 9-month PFS. This number of patients would also be sufficient to demonstrate non-inferiority of the extended frequency arm, using a hazard ratio non-inferiority margin of 1.94 for the renal cohort (and e.g. 1.85 for the melanoma cohort) which should correspond to a 20-percentage points NI margin on the 9-month PFS difference scale. 9-month PFS rates are estimated to be 70% in the renal cohort [1,18]. For all cohorts, stage I analysis will be performed when approximately 30 PFS events have been observed in the control arm, which is expected to occur around six months after the completion of recruitment.

Following analysis of stage I data, assuming satisfactory outcomes in patients treated with the extended dosing interval, stage II will then test multiple experimental arms with the maximal interval being 3 times the SOC. The second stage intends to enrol up to an additional 200 patients per cohort dependent on stage I results. Under the same assumptions made for the stage 1 sample size calculations, this will provide around 80% power to demonstrate non-inferiority for the extended interval arm, relative to SOC using the same non-inferiority margins.

### 2.4 Health economic considerations

The economic evaluation for stage I of the trial will evaluate the relative cost-effectiveness of the two treatment frequencies over a 12-month period. The incremental cost per quality-adjusted life-year (QALY) gained will be calculated as recommended by the National Institute for Health and Care Excellence (NICE) [19], using QALYs calculated from EQ-5D-5L responses collected at baseline and every 12 weeks, including after progression [20], from the perspective of the NHS and Personal Social Services.

Resource use will be collected at baseline for the preceding 12 weeks and every 12 weeks thereafter from patient records and directly from participants via a modified version of the Client Service Receipt Inventory (CSRI) [21], covering inpatient and outpatient hospital service use, urgent care, primary and community health and social care service contacts, and therapies including ICI treatment costs, radiotherapy, surgery, and other systemic cancer treatments. Costs involved in administering ICI will be included in both arms.

Parameter uncertainty will be addressed using standard boot-strapping techniques and sensitivity analyses performed for any assumptions made. Further details will be described in the statistical and health economic analysis plan.

A lifetime decision analytic model will be constructed using information collected in stage I and stage II for those arms that are carried forward, to estimate the relative cost-effectiveness of the different treatment frequencies.

### 2.5 Eligibility and participant recruitment

Participants entering REFINE will be patients with locally advanced or metastatic cancer receiving ICI as SOC and responding to treatment (complete/partial response or stable disease radiologically) at 12 weeks in the UK. They must satisfy the eligibility criteria summarised in Table 1 and 2. Randomisation can occur anytime up to 6 weeks after the last dose of the first 12 weeks of treatment.

### 2.6 Site recruitment

REFINE is open to NHS hospitals throughout the United Kingdom (UK). Site recruitment has been organised in ‘waves’ of hospitals, grouped by their accrual to RAMPART (a phase III trial platform trial of adjuvant therapy in high/intermediate risk RCC) and PRISM (a randomised phase II trial of nivolumab with alternatively scheduled ipilimu-mab in advanced/metastatic RCC) [22,23]. This has enabled more individualised site support and enabled the optimisation of training and materials provided to hospital sites. Information for sites is regularly updated on the trial website: http://refine.mrcctu.ucl.ac.uk/. All participating sites will be managed by the MRC CTU at UCL.

### 2.7 Randomisation

REFINE will randomise participants centrally through an interactive web-based system using minimisation (with a random element) with stratification by a small number of important stratification factors. For stage I, patients will be randomly assigned in the ratio 1:1 to Arm A (SOC interval) and Arm B (extended interval). The precise allocation ratio for stage II will be determined after completion of stage I, but it is anticipated that more patients will be allocated to the added arms than to the existing stage I arms, to achieve approximately equal total allocation between all arms.

### 2.8 Co-enrolment

Participants previously enrolled in interventional trials may be eligible for recruitment to REFINE, if they have not received prior ICI. REFINE participants should not join any other interventional trial during treatment in REFINE, unless they have experienced disease progression. Co-enrolment in non-interventional studies is permitted if it does not interfere with the REFINE treatment/assessment schedule.

### 2.9 Treatment schedule and assessments

Participants in Arm A will receive SOC interval ICI (480 mg nivo-lumab 4 weekly after ipilimumab and nivolumab regimens) commencing 6 weeks after their last infusion prior to randomisation.

Participants in Arm B will receive extended interval ICI, which in stage I is doubling the dosing interval of Arm A (SOC interval):

Additional tumour cohorts will mirror the renal cohort if treated with ipilimumab/nivolumab combinations (e.g. patients with melanoma) randomised at the point they proceed to nivolumab monotherapy. If treated with pembrolizumab (e.g. melanoma or mismatch repair-deficient Colorectal Cancer) with a SOC of 400 mg pem-brolizumab 6 weekly, the experimental arm for stage I will be 12 weekly administration.

Treatment will be given as part of the trial until disease progression, toxicity that precludes further treatment, participant preference or for 1 year 9 months from the start of randomised treatment (i.e. 2 years total treatment). Participants who are still responding to ICI treatment at 2 years will have the option to continue treatment off trial if part of their normal care. Participants experiencing progression on the extended dosing interval (Arm B) will be offered treatment intensification (i.e. reverting to SOC ICI dosing frequency, as per Arm A) at the participants and clinician's discretion.

The trial assessment schedule for each cohort is aligned with standard practice. Participants will be clinically assessed at baseline and every 4 weeks for the trial duration regardless of treatment status or progression. Since the COVID-19 pandemic clinical assessments are often conducted remotely, and the REFINE assessment schedule supports this, if possible, to collect all necessary clinical information and this is deemed appropriate by the investigator.

Participants in both arms have a CT with contrast of the chest, abdomen and pelvis (or Positron Emission Tomography (PET)-CT +/− brain surveillance if appropriate in the melanoma cohort) carried out at baseline and then every 12 weeks until disease progression or treatment discontinuation, whichever occurs later.

### 2.10 Treatment beyond progression

Participants will be permitted to continue treatment beyond initial progression if they meet the following criteria:

- Investigator-assessed clinical benefit
- Tolerance of study drug
- Stable performance status

Participants on Arm B (extended interval ICI) who experience progression and are clinically fit to continue treatment may continue the extended interval dosing or will have the option to intensify their treatment (i.e., cross over back to Arm A regimen). ICI treatment should be discontinued permanently upon documentation of further progression.

### 2.11 Criteria for discontinuing allocated interventions

An individual participant may stop treatment early for any of the following reasons:

- Disease progression
- Unacceptable toxicity, intercurrent illness or change in patient's condition that justifies discontinuation
- Inadequate compliance with the protocol treatment, in the judgement of the treating physician
- Pregnancy or intent to become pregnant
- Grade ≥ 3 infusion reaction
- Initiation of alternative anticancer therapy including another investigational agent
- Any dosing interruption lasting >6 weeks although dosing delays or interruptions to allow for prolonged steroid tapers to manage drug-related adverse events are allowed.
- Withdrawal of consent for treatment by the patient

### 2.12 Participant follow up

Participants will be followed up for 1 year and 9 months from randomisation, regardless of treatment status or progression. Longer term data including information on progression and survival will be collected on an annual basis until the end of the trial.

### 2.13 Tumour assessment

PFS outcome will be investigator-reported and will be based on radiological progression according to RECIST 1.1 criteria. Radiology reports will be collected and reports on progression will be reviewed blinded to treatment allocation.

### 2.14 Safety/toxicity management

ICI stimulate a host immune response against tumour cells. They can also lead to activation of self-reactive T cells that can damage healthy tissue and result in immune related adverse events (ir-AEs) which can affect any body system. A current list of ir-AEs is available to investigators in the Reference Safety Information (RSI). ir-AEs should be managed as per local and national/international guidelines.

### 2.15 Duration

It is expected that recruitment to stage I will complete in approximately 12 months. Analysis of disease control will be performed at approximately 6 months from completion of stage I recruitment. As the results of stage I will inform which arms are included in stage II, during this time randomisations will be paused. It is then expected that recruitment to stage II will complete in a further 12 months. Analysis of disease control will be performed within 12 months of recruitment completion.

### 2.16 Monitoring

The monitoring plan for REFINE is based on a formal risk assessment and reviewed and updated as appropriate on at least an annual basis. The REFINE team conduct monitoring checks including consent, eligibility, treatment administration and safety monitoring. Any issues identified will be raised and discussed with the local team. Central monitoring processes have been expanded where possible to replace some of the activities that would normally be completed during on-site monitoring visits in light of the Covid 19 pandemic. On-site monitoring for the REFINE trial will be conducted on a triggered basis (triggers include low data return rate, poor data quality, late reporting on SAEs and critical protocol deviations). Any sites meeting at least 2 of the triggers will be considered for an on-site monitoring visit. The triggers will be reviewed by the trial team at least annually.

### 2.17 Data management

Electronic CRFs are used in REFINE. Sites enter data directly into the REFINE Open Clinica Database. Built in validations (value ranges, date inconsistencies, treatment administration) help ensure the data is correct. The REFINE Data Management Plan provides a breakdown of how data is to be acquired, handled, and secured.

### 2.18 Statistical analysis plan

For stage I, the primary analysis will consist of a Cox proportional hazards model, where time will be time to progression or censoring, and the main covariate will be binary treatment, defined as standard-of-care (Arm A – standard frequency) or extended frequency (Arm B) ICI. The model will also adjust for the stratification variables. Each cohort will be analysed separately, using a 5% one-sided significance level. All patients will be included and analysed by their schedule allocation (intention to treat – ITT). The hazard ratio for Arm B vs Arm A will then be estimated and will inform the design of stage II, following discussion with relevant oversight committees (Trial Management Group (TMG), Independent Data Monitoring Committee (IDMC) and Trial Steering Committee (TSC)). Strict statistical rules will not be in place, but decisions will be based on the following guidelines:

- If the stage I analysis shows that the extended interval arm (2×) is significantly inferior to the standard interval arm (1×), i.e. the confidence interval (CI) for the hazard ratio lies entirely above 1, recruitment will not be continued in the 2× arm, and no arms with further extended intervals (2.5× or 3×) will be opened. The trial committees will discuss whether there is any scientific merit in opening the intermediate interval (1.5×) arm (Fig. 1).
- If the extended frequency arm is not shown to be significantly inferior, an assessment of non-inferiority will be made. If non-inferiority is shown, (CI for the hazard ratio excludes the non-inferiority margin), then stage II can proceed as planned, opening up arms with further extended intervals (2.5× and 3×). The trial committees will further discuss whether there is merit in adding the intermediate interval (1.5×) arm at this stage.
- In the event that the stage I results show neither inferiority or noninferiority of the extended frequency arm, (CI for the hazard ratio includes both 1 and the non-inferiority margin), the trial committees will determine which arms will be available in stage II. Safety and quality of life data will be considered alongside the efficacy data, as well as any emerging external evidence, including information from other REFINE cohorts.

Decisions will be made independently for each cohort; not all cohorts may continue to stage II, and different arms may be introduced in each cohort.

Analysis of PFS in stage II will be performed as described for stage I, fitting a Cox model for PFS and estimating hazard ratios comparing extended frequency arms to the standard frequency arm. A 5% (one-sided) significance level will be used throughout. Analysis will begin by assessing the most extended frequency arm. If that arm demonstrates non-inferiority, no further arms will be compared, and all arms will be considered for the phase III expansion of REFINE. If non-inferiority is not demonstrated, the next most extended frequency arm is assessed in the same manner, and so on until either an arm demonstrates non-inferiority, or all arms have been assessed.

If there is evidence of non-proportional hazards, or if other characteristics of the data suggest a likely gain in power, restricted mean survival (RMST) with a t-star of nine months will be used to analyse these data instead [24].

For time to event analyses, patients without an observed event will be censored at the date of their last assessment. It is anticipated that the majority of censoring will be administrative censoring at the time of analysis, therefore the assumption of non-informative censoring should be valid. Patterns of censoring will be assessed and if necessary, for example if a high proportion of patients are lost to follow-up, sensitivity analyses may be performed to assess the robustness of results.

Analysis of categorical variables will compare arms using the chi-squared or Mann-Whitney test, with analysis of continuous variables using a *t*-test. Continuous secondary outcome measures such as QOL may utilise linear mixed models to obtain an estimate for the mean difference between treatment arms. Models will include a random intercept, plus arm, time-by-arm interaction and randomisation stratification variables as fixed effects. Model assumptions will be examined using residual analysis and examination of graphical displays such as normal quantile plots. For quality-of-life analyses, initial analyses will use a complete case approach, without imputation of any missing data. Levels and patterns of missing data will be assessed, and if necessary, sensitivity analyses will be conducted using multiple imputation techniques.

All estimates will be presented along with their 95% confidence interval. Additionally, to assess non-inferiority, PFS hazard ratios will also include a 90% confidence interval, corresponding to the 5% one-sided significance interval. All disease cohorts are designed to be independently powered and analysed.

All analyses will be conducted using the intention-to-treat (ITT) population, including all randomised patients. The primary outcome measure of PFS will also be assessed using the per-protocol (PP) population, which will exclude any ineligible patients, and any patients who do not receive any treatment after randomisation.

### 2.19 Translational sub-studies

Translational work within REFINE aims to understand the pharmacokinetic and pharmacodynamic aspects of extended versus standard interval ICI, conducted in collaboration with Imperial College London and the US National Cancer Institute (NCI). Serial blood samples will be collected from approximately 80 patients (40 from SOC and 40 from extended interval) during their first 9 months on study. Pharmacokinetic analysis of therapeutic drug levels and flow cytometry to determine T cell receptor occupancy will determine whether extended interval dosing influences T cell activation. Consent for this sub- study is optional.

### 2.20 Regulatory and ethical considerations

The trial will be conducted in compliance with the approved protocol, the Declaration of Helsinki 2008, the principles of Good Clinical Practice (GCP) as laid down by the ICH topic E6 (R2), Commission Clinical Trials Directive 2005/28/EC* with the implementation in national legislation in the UK by Statutory Instrument 2004/1031 and subsequent amendments, General Data Protection Regulation and the UK Data Protection Act 2018, and the UK Policy Framework for Health and Social Care Research.

The Medicines and Healthcare Products Regulatory Agency (MHRA) granted Clinical Trials Authorisation on the 6th September 2021. The Westminster Research Ethics Committee granted ethical approval on 19th October 2021.

### 2.21 Patient and public involvement

Patient and public involvement (PPI) representatives are involved in all aspects of REFINE including initial concept and trial design, patient facing materials and how to maximise recruitment. They are represented on the TMG and TSC and will be actively involved in discussions on trial progress including reviewing IDMC recommendations.

### 2.22 Trial oversight

REFINE is sponsored by UCL. The MRC CTU at UCL has overall responsibility for the study working closely with the Chief Investigator (CI), all members of the TMG and all collaborators. The Trial Management Team (TMT), comprising CI, clinical project manager, trial manager, data manager and statistician, meet weekly to discuss all aspects of trial management including site set up, recruitment and safety reporting. The TMT report to the TMG who oversee the conduct of the trial and meet formally every 6 months. The IDMC meet annually to review unblinded accumulating efficacy and safety data. The IDMC and TMG report to the TSC who provides overall supervision of the trial.

## 3 Conclusion

REFINE is a randomised, phase II, MAMS adaptive basket trial investigating extended interval administration of ICI across multiple cancer types. Eligible patients are those responding (radiologically) to ICI at 12 weeks. The REFINE RCC cohort opened to recruitment on the 25th May 2022 and the first patient was randomised on 22nd August 2022. Subsequent cohorts will be added in the next 12 months. Stage I will recruit 160 patients to each cohort who will be randomised to SOC versus extended dose interval. Disease control will be analysed 6 months from completion of stage I recruitment, and this will inform stage II which will investigate up to 5 different treatment intervals. The REFINE Translational sub-study will investigate the pharmacokinetic and pharmacodynamic aspects of extended interval ICI treatment and health economic evaluation will evaluate the cost effectiveness of extended interval ICI.

Completion of the first 2 stages of REFINE will inform the design of a phase III trial testing extended dosing interval of administration of different ICI in responding patients across multiple indications. REFINE is being run in parallel with REFINE-LUNG, a phase III trial evaluating extending the interval of pembrolizumab in advanced non-small-cell-lung-cancer(NSCLC) [25] which is funded by the National Institute of Health and Care Research (NIHR) and co-ordinated by Imperial College.

Extending the interval of ICIs has potential benefits in improving patients' quality of life, via both reduced side effects and fewer hospital visits, and in more efficient use of healthcare services via lower drug treatment and attendance costs. REFINE and REFINE-Lung have joined a growing initiative of studies aiming to optimise treatment with ICI and these studies should be supported [26]. Reducing costs and the burden associated with treatment may improve access to ICI, allowing the global population to benefit from their transformative cancer outcomes.

An up-to-date version of the REFINE protocol can be found at: http://refine.mrcctu.ucl.ac.uk/.

## Figures and Tables

**Table 1:** Summary of inclusion criteria (full list available on REFINE website [27]).

Inclusion Criteria – General
• Patients with locally advanced or metastatic cancers for which ICIs are SOC
• WHO Performance Status 0 or 1
• ≥18 years of age
• Adequate normal organ and marrow function:
a) Haemoglobin ≥90 g/L
b) Neutrophil count ≥1.5 × 109/L
c) Platelet count ≥100 × 109/L
d) Bilirubin ≤1.5 × ULN
e) Aspartate aminotransferase/Alanine Aminotransferase ≤3 × ULN
f) eGFR >40 mL/min
• Resting 12-lead ECG with corrected QT interval <450 ms
• Following trial's contraception procedure
• Evidence of post-menopausal status or negative serum human chorionic gonado-trophin (HCG) pregnancy test for female pre/peri-menopausal patients
Inclusion Criteria – Cohort Specific
Renal
• Patients with unresectable locally advanced or metastatic renal cell carcinoma
(including clear cell and papillary histology)
• Intermediate or poor risk as defined in the International Metastatic Renal Cell
Carcinoma Database Consortium criteria (prior to the initial 12 weeks treatment
with ICI combination)
• No evidence of progression on ipilimumab and nivolumab induction therapy and
due to commence maintenance nivolumab
Melanoma
• Patients with locally advanced or metastatic melanoma.
• No evidence of progression on ipilimumab and nivolumab induction therapy and
due to commence maintenance nivolumab
OR
• Patients have received single agent pembrolizumab first line for 12 weeks, with no
evidence of progression and are due to commence maintenance pembrolizumab
every 6 weeks

**Table 2:** Summary of exclusion criteria for REFINE (full list available on REFINE website [27]).

Exclusion criteria
• Patients who have received ICI in a prior line of treatment
• Patients who have undergone any prior systemic anti-cancer treatment
• Patients where treatment is the combination of anti-PD-1 and tyrosine kinase inhibitor or the combination of cytotoxic chemotherapy and anti-PD-1
• History of another previous malignancy (some exceptions apply)
• Concurrent enrolment in another interventional clinical study, unless in the follow-up period, except where approved by the CTU
• Current or prior use of immunosuppressive medication within 14 days of starting trial treatment, with the exceptions of intranasal and inhaled corticosteroids or systemic corticosteroids at physiological doses, which are not to exceed 10 mg/day of prednisone, or an equivalent corticosteroid
• Active infection including Tuberculosis, Hepatitis B, Hepatitis C and HIV
• Patients who have received a live attenuated vaccine within 30 days prior to the start of treatment
• Known allergy or hypersensitivity to immune checkpoint inhibitor
• Pregnant or breastfeeding patients
• Uncontrolled adrenal insufficiency
• Any serious or uncontrolled medical or psychiatric disorder that, in the opinion of the investigator, may increase the risk associated with study participation
• Untreated brain metastases or brain metastases treated only with whole brain radiotherapy

## Data Availability

No data was used for the research described in the article.
